# Secukinumab in enthesitis-related arthritis and juvenile psoriatic arthritis: a randomised, double-blind, placebo-controlled, treatment withdrawal, phase 3 trial

**DOI:** 10.1136/ard-2022-222849

**Published:** 2022-08-12

**Authors:** Hermine I Brunner, Ivan Foeldvari, Ekaterina Alexeeva, Nuray Aktay Ayaz, Inmaculada Calvo Penades, Ozgur Kasapcopur, Vyacheslav G Chasnyk, Markus Hufnagel, Zbigniew Żuber, Grant Schulert, Seza Ozen, Adelina Rakhimyanova, Athimalaipet Ramanan, Christiaan Scott, Betul Sozeri, Elena Zholobova, Ruvie Martin, Xuan Zhu, Sarah Whelan, Luminita Pricop, Alberto Martini, Daniel Lovell, Nicolino Ruperto

**Affiliations:** 1 UC Department of Pediatrics, University of Cincinnati, Cincinnati Children's Hospital Medical Center, Cincinnati, Ohio, USA; 2 Hamburger Zentrum fuer Kinder und Jugendrheumatologie, Hamburg, Germany; 3 National Scientific and Practical Center of Children's Health, Ministry of Health of the Russian Federation, Moscow, Russian Federation; 4 Istanbul University-Cerrahpasa, Faculty of Medicine, Department of Pediatric Rheumatology, Istanbul, Turkey; 5 Hospital Universitario y Politécnico La Fe Valencia, Reumatologìa, Valencia, Spain; 6 Pediatric Rheumatology, Istanbul Universitesi-Cerrahpasa, Istanbul, Turkey; 7 State Pediatric Medical University, Department of Pediatric Rheumatology, Saint-Petersburg, Russian Federation; 8 University Medical Center, Medical Faculty University of Freiburg, Department of Pediatrics and Adolescent Medicine, Freiburg, Germany; 9 Andrzej Frycz Modrzewski Krakow University, Faculty of Medicine and Health Sciences, Department of Pediatrics, Krakow, Poland; 10 UC Department of Pediatrics, Cincinnati Children's Hospital Medical Center, Cincinnati, Ohio, USA; 11 Department of Pediatrics, Hacettepe University, Ankara, Turkey; 12 Regional Children Clinical Hospital # 1, Ural State Medical University, Ministry of Healthcare of the Russian Federation, Department of Rheumatology, Yekaterinburg, Russian Federation; 13 University Hospitals Bristol NHS Foundation Trust, Bristol, UK; 14 University of Bristol, Department of Pediatric Rheumatology, Bristol, UK; 15 Red Cross War Memorial Children’s Hospital, University of Cape Town, Department of Pediatric Rheumatology, Cape Town, South Africa; 16 Umraniye Training and Research Hospital, Department of Pediatric Rheumatology, Istanbul, Turkey; 17 First Moscow State Medical University n.a. I.M.Sechenov, Department of Rheumatology, Moscow, Russian Federation; 18 Novartis Pharmaceuticals Corporation, East Hanover, New Jersey, USA; 19 Novartis Ireland Ltd, Dublin, Ireland; 20 Università degli Studi di Genova, Genova, Italy; 21 Rheumatology, Cincinnati Children's Hospital Medical Center, Cincinnati, Ohio, USA; 22 IRCCS Istituto Giannina Gaslini, UOSID Centro Trial, Genova, Italy

**Keywords:** biological therapy, arthritis, juvenile, arthritis, psoriatic

## Abstract

**Background:**

Treatment options in patients with enthesitis-related arthritis (ERA) and juvenile psoriatic arthritis (JPsA) are currently limited. This trial aimed to demonstrate the efficacy and safety of secukinumab in patients with active ERA and JPsA with inadequate response to conventional therapy.

**Methods:**

In this randomised, double-blind, placebo-controlled, treatment-withdrawal, phase 3 trial, biologic-naïve patients (aged 2 to <18 years) with active disease were treated with open-label subcutaneous secukinumab (75/150 mg in patients <50/≥50 kg) in treatment period (TP) 1 up to week 12, and juvenile idiopathic arthritis (JIA) American College of Rheumatology 30 responders at week 12 were randomised 1:1 to secukinumab or placebo up to 100 weeks. Patients who flared in TP2 immediately entered open-label secukinumab TP3 that lasted up to week 104. Primary endpoint was time to disease flare in TP2.

**Results:**

A total of 86 patients (median age, 14 years) entered open-label secukinumab in TP1. In TP2, responders (ERA, 44/52; JPsA, 31/34) received secukinumab or placebo. The study met its primary end point and demonstrated a statistically significant longer time to disease flare in TP2 for ERA and JPsA with secukinumab versus placebo (27% vs 55%, HR, 0.28; 95% CI 0.13 to 0.63; p<0.001). Exposure-adjusted incidence rates (per 100 patient-years (PY), 95% CI) for total patients were 290.7/100 PY (230.2 to 362.3) for adverse events and 8.2/100 PY (4.1 to 14.6) for serious adverse events in the overall JIA population.

**Conclusions:**

Secukinumab demonstrated significantly longer time to disease flare than placebo in children with ERA and JPsA with a consistent safety profile with the adult indications of psoriatic arthritis and axial spondyloarthritis.

**Trial registration number:**

NCT03031782.

**WHAT IS ALREADY KNOWN ON THIS TOPIC**

- Treatment options for patients with the juvenile idiopathic arthritis (JIA) subtypes of enthesitis-related arthritis (ERA) and juvenile psoriatic arthritis (JPsA) are limited. Conventional synthetic disease-modifying antirheumatic drugs (DMARDs), glucocorticoids and non-steroidal anti-inflammatory drugs provide limited efficacy with safety issues with long-term use. Studies have shown the efficacy of biologic DMARD (bDMARD) anti-TNF agents in patients with ERA or JPsA, but many patients continue to experience uncontrolled disease or experience treatment-related side effects.

**WHAT THIS STUDY ADDS**

- In this double-blind, randomised, placebo-controlled, event-driven treatment withdrawal phase 3 study, the time to JIA disease flare was statistically longer in patients treated with secukinumab compared with placebo-treated patients from week 12 up to week 104 (27% vs 55%, HR, 0.28; 95% CI 0.13 to 0.63; p<0.001).
- Improvements in JIA American College of Rheumatology (ACR) responses, JIA ACR-inactive disease, JIA ACR core set components, Juvenile Arthritis Disease Activity Score were reported in patients treated with secukinumab up to 12 weeks.
- No new safety signals were reported with secukinumab for up to 2 years.

**HOW THIS STUDY MIGHT AFFECT RESEARCH, PRACTICE OR POLICY**

- The results from this study support that secukinumab, a fully human monoclonal antibody that inhibits IL-17A, is an effective and safe bDMARD in the treatment of patients with ERA and JPsA categories of JIA. Safety profile of secukinumab in JIA categories was consistent with reported adult indications of psoriatic arthritis and axial spondyloarthritis.

## Introduction

Juvenile idiopathic arthritis (JIA) is a heterogeneous group of inflammatory disorders that includes patients with arthritis of unknown aetiology that starts before the age of 16 years and persists for 6 or more weeks.^1^ Enthesitis-related arthritis (ERA) and juvenile psoriatic arthritis (JPsA) are two categories of JIA that represent paediatric counterparts of adult non-radiographic axial spondyloarthritis (nr-axSpA) and psoriatic arthritis (PsA), respectively.^1 2^

Non-steroidal anti-inflammatory drugs (NSAIDs) and conventional synthetic disease-modifying anti-rheumatic drugs (csDMARDs) are considered first-line agents in JPsA, while NSAIDs and sulphasalazine are considered for ERA. Although the current treatment strategies for ERA and JPsA help to relieve pain, these medications often provide limited efficacy on the underlying disease.^3^ This necessitates initiation of more intensive therapy, including the introduction of biologic (b)DMARDs, of which very few are approved for ERA and JPsA treatments.^4 5^

The interleukin (IL)−17A pathway plays an important role in the pathogenesis of ERA and JPsA. Compared with controls, increased levels of IL-17A were reported in patients with JIA, especially in the setting of active disease.^6^ Progression of structural damage is mediated by the IL-17 pathway in inflammatory arthritis.^7^ Secukinumab, a fully human monoclonal antibody that directly inhibits IL-17A, has demonstrated efficacy and safety in adult patients with psoriasis (PsO), PsA, ankylosing spondylitis (AS) and nr-axSpA.^8–11^ This phase 3 study demonstrated the efficacy and safety of secukinumab in patients with active ERA and JPsA.

## Methods

### Study design and participants

This was a 2-year, randomised, double-blind, placebo-controlled, event-driven, treatment-withdrawal, phase 3 study that consisted of three treatment periods (TPs): open-label (OL) secukinumab TP1 (up to 8 weeks); a randomised, double-blind, placebo-controlled, withdrawal period (up to week 104) TP2; OL secukinumab TP3 and a post-treatment follow-up period (online supplemental figure S1). The study was planned to be continued until 33 flare events had occurred or randomised patients had completed 104 weeks in the study. In this event-driven, treatment-withdrawal study, the patients were treated first in an OL manner with the study drug for 12 weeks, and responders (JIA American College of Rheumatology (ACR)30 responders) were randomised to the study drug or placebo in a double-blind manner. In this phase of treatment, occurrence of a protocol-defined disease flare led to withdrawal from the double-blind treatment phase and patients were retreated with the study drug in an OL fashion.^12^

10.1136/ard-2022-222849.supp1 Supplementary data

**Figure 1:**
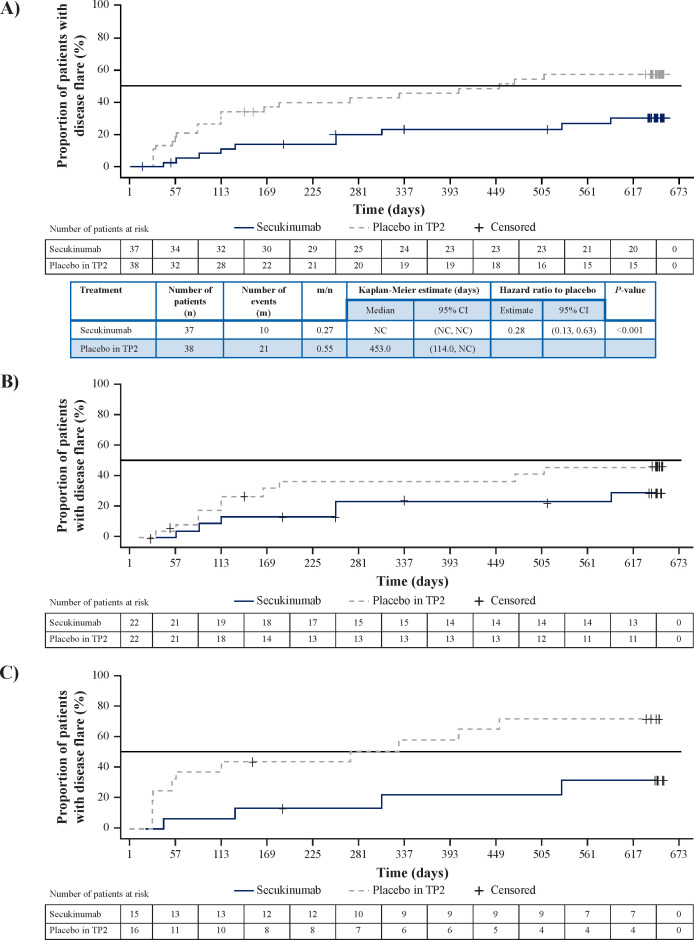
Time to disease flare in (A) overall JIA population and JIA subcategories of (B) ERA and (C) JPsA in TP2 A) JIA: 72% flare risk reduction; HR, 0.28; 95% CI, 0.13 to 0.63; p<0.001 B) ERA: 55% flare risk reduction; HR, 0.45; 95% CI, 0.16 to 1.28; p=0.075 C) JPsA: 85% flare risk reduction; HR, 0.15; 95% CI, 0.04 to 0.57; p<0.001. HR and associated 95% CIs were based on a Cox proportional hazards model with treatment and analysis factors JIA category (ERA or JPsA) and MTX use at baseline as explanatory variables. Log-rank test was adjusted for analysis factors JIA category (ERA or JPsA) and methotrexate use at baseline. SEC: all patients who did not take any placebo. Placebo in TP2: all patients who took placebo in TP2 and SEC in other period/s. Day 1: date of randomisation. Disease flare was derived relative to the end of TP1 (week 12 visit). Patients who did not experience a disease flare in TP2, were censored at the date of their last non-missing flare evaluation in TP2. Patients at risk: patients in TP2 who did not have flare and were not censored before or at the start of the specified day. ERA, enthesitis-related arthritis; JIA, juvenile idiopathic arthritis; JPsA, juvenile psoriatic arthritis; m/n, proportion of patients with disease flare in TP2; NC, not calculable; SEC, secukinumab; TP, treatment period.

Key inclusion criteria included patients aged ≥2 to <18 years at screening, classified according to the International League of Associations for Rheumatology JIA classification criteria as either ERA or JPsA, disease duration of ≥6 months with inadequate response to ≥1 csDMARDs or NSAIDs. Eligible patients were naïve to bDMARDs and had active disease. Key exclusion criteria included active uncontrolled inflammatory bowel disease (IBD) or uncontrolled uveitis. Detailed inclusion and exclusion criteria are provided in online supplemental appendix (Section S2).

Eligible patients entered TP1 and received OL subcutaneous secukinumab in prefilled syringes (75/150 mg in patients <50/≥50 kg). During TP1, secukinumab was administered weekly up to week 4, followed by every 4 weeks (Q4W). At week 12 (end of TP1), JIA ACR30 responders entered TP2 and were randomised 1:1 to continue secukinumab or receive placebo Q4W until a disease flare, or up to week 100. JIA ACR30 non-responders at week 12 were discontinued from the study. All patients who experienced a disease flare in TP2 or completed TP2 without a disease flare entered TP3 to again receive OL secukinumab Q4W up to week 100.

The study protocol was reviewed and approved by the respective ethics committee or institutional review board of each centre. The study was conducted according to the International Council for Harmonization Good Clinical Practice guidelines.

### Randomisation

Eligible patients were randomised (1:1) to continue secukinumab or newly start placebo in TP2. Randomisation was performed centrally using an Interactive Response Technology system and was stratified by JIA category (ERA, JPsA) (Section S3, online supplemental appendix).

### Procedures

Secukinumab was administered subcutaneously in prefilled syringes (75/150 mg in patients <50/≥50 kg). Study visits and study drug administration were scheduled at baseline and weeks 1, 2, 3 and 4, followed by 4 weekly visits through week 104 with study drug administration until week 100. Patients could continue stable background therapies with NSAIDs, methotrexate,^13^ sulfasalazine and oral glucocorticoids (Section S4, online supplemental appendix). Efficacy outcomes and adverse events (AEs) were assessed at each study visit.

### Outcomes and assessments

All study outcomes are listed in the study protocol, which is provided in the online supplemental appendix (Section S5). At each planned visit, data for the six validated JIA ACR core set variables (CRVs) were recorded.^14^ The JIA ACR30 response as per the JIA ACR response criteria is defined as ≥30% improvement in three or more of six CRVs, with no more than one of the remaining CRVs worsening by >30%.^14^ The primary end point was time to JIA flare in TP2, defined as per the Pediatric Rheumatology Collaborative Study Group/Paediatric Rheumatology INternational Trials Organisation JIA flare criteria as ≥30% worsening from baseline in at least three of the six CRVs with no more than one of the remaining CRVs with >30% improvement relative to the end of TP1 (week 12).^15^ Key secondary efficacy end points included JIA ACR30/50/70/90/100 responses, inactive disease (JIA ACR-ID) status^16^ JIA ACR CRVs, Juvenile Arthritis Disease Activity Score (JADAS)−27-C reactive protein and total enthesitis and dactylitis counts at week 12 in TP1, and JIA ACR30/50/70/90/100 responses and JIA ACR-ID status at the end of TP2.

Safety analyses were conducted for the entire study period (TP1–TP3) in the overall JIA population. Safety assessments included all AEs coded as per the *Medical Dictionary for Regulatory Activities* (V.23.1), serious AEs (SAEs), treatment-emergent AEs (TEAEs), injection site reactions and antisecukinumab antibody development (immunogenicity).

### Statistical analysis

For sample size estimation, the HR of flare events for the secukinumab group relative to the placebo group was estimated to be 0.32 in TP2.^17^ Thirty-three flares (12 for secukinumab, 21 for placebo) were estimated to detect a statistically significant difference between secukinumab and placebo to achieve 90% power by an one-sided significance level of 0.025. With an event-driven approach, the study was to be either stopped once 33 disease flares were detected or last patient’s last visit over the study is achieved. We expected that a maximum 92 weeks of TP2 was necessary to observe 33 disease flares. Assuming approximately 85% of the patients respond at JIA ACR30 levels in TP1, we estimated that at most 94 patients needed to be enrolled into the study to allow for a sufficient number of patients to be randomised into TP2.

To show the superiority of secukinumab over placebo on the primary end point of time to disease flare in the two treatment groups, an one-sided stratified log-rank test was used with treatment, stratification variable of JIA category (ERA or JPsA) and methotrexate use as explanatory variables. HR and their associated 95% CIs were estimated based on a Cox proportional hazards model. Kaplan-Meier estimates (95% CI) of the probability of disease flare by treatment groups were calculated. Patients either experienced a disease flare or were censored in TP2. Subgroup analyses for time to disease flare were performed for the stratification variable of JIA category (ERA or JPsA). The intention-to-treat principle was applied for all primary and key secondary efficacy analyses.

The safety data analysis was conducted on the safety set that included all patients who received at least one dose of secukinumab. Exposure-adjusted incidence rates (per 100 patient-years (PY) of follow-up) were calculated for AEs and SAEs. An independent data safety monitoring board was responsible for ongoing monitoring of the safety of patients in the study.

### Patient and public involvement

Patients or the public were not involved in the design or conduct of the trial. Written informed consent was obtained from parents or legal guardians of each patient at screening.

## Results

### Patient characteristics

Between 23 May 2017 and 9 November 2020, among 97 patients screened for eligibility, 86 patients (88.7%) entered TP1 to receive OL secukinumab (online supplemental figure S2). There were 52 patients (60.5%) diagnosed with ERA and 34 (39.5%) with JPsA.

**Figure 2:**
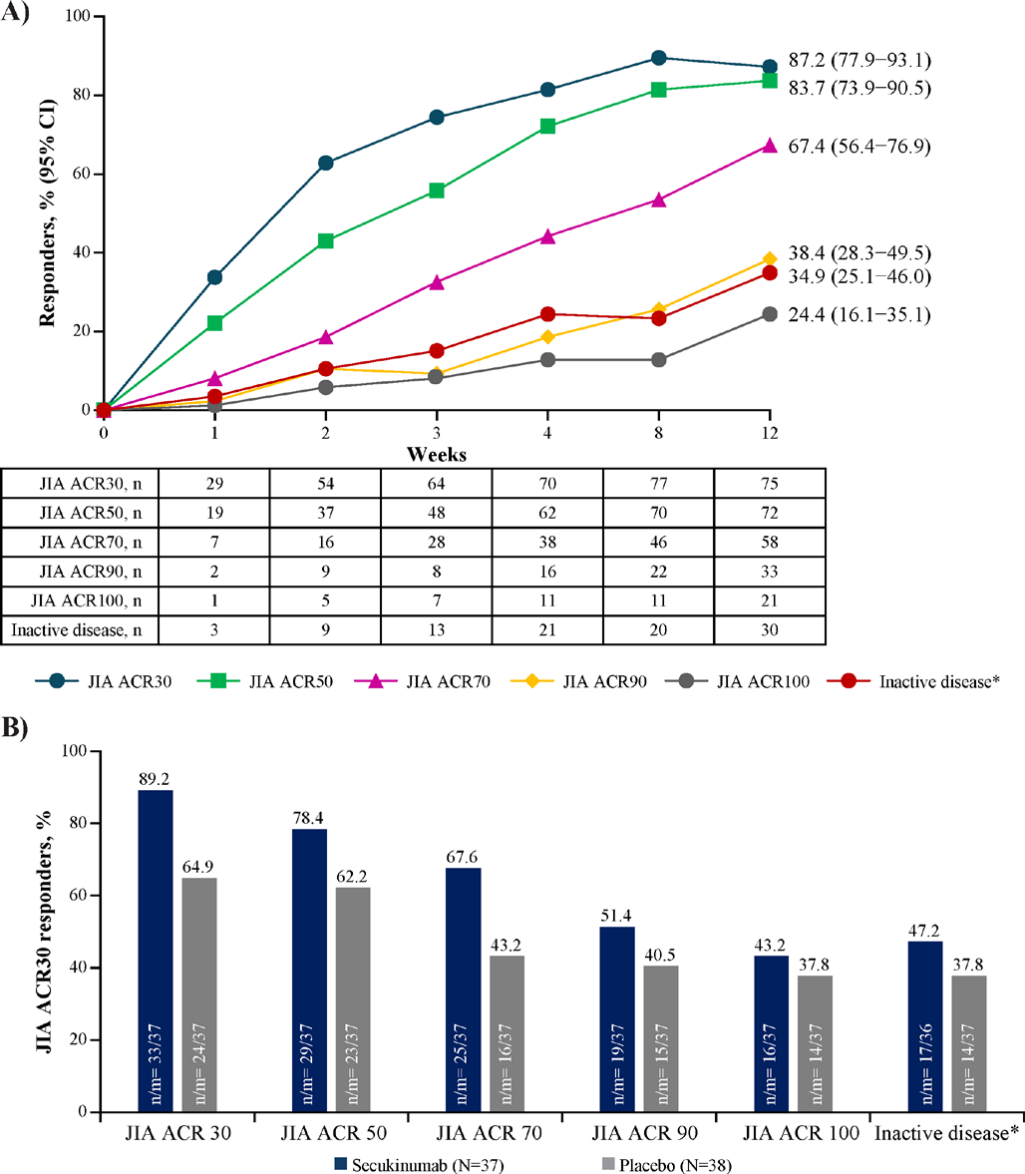
Improvement in ACR responses from baseline with open-label secukinumab in TP1 (A), and improvement from baseline at the end of TP2 (B). ^*^Inactive disease status. 95% CI are from NRI analysis of ACR response derived relative to baseline. N=86 N, number of patients in the full analysis set; n, number of patients with response (B). *Inactive disease status. NRI analysis. ACR, American College of Rheumatology; JIA, juvenile idiopathic arthritis; m, total number of patients with an assessment at the end of TP2; N, total number of patients in the treatment group; n, number of patients who satisfy the criteria; NRI, non-responder imputation; TP, treatment period.

At baseline, there was a male preponderance in the ERA group (78.8%, 41/52) and a slight female majority in the JPsA group (52.9%, 18/34) (table 1). Of the 86 patients enrolled in TP1, 75 patients (87.2%) with a JIA ACR30 response at week 12 (end of TP1) were allowed to enter TP2. The baseline characteristics and clinical features of patients who entered TP2 were comparable to those of the overall study cohort (online supplemental table S1).

**Table 1:** Baseline demographics and clinical characteristics of all JIA patients and each JIA category in TP1

Variable	JIA (N=86)	ERA (N=52)	JPsA (N=34)
Age (years), mean (SD)	13.1 (3.1)	13.7 (2.6)	12.2 (3.7)
Male, n (%)	57 (66.3)	41 (78.8)	16 (47.1)
Race, n (%)			
White	82 (95.3)	51 (98.1)	31 (91.2)
Other*	4 (4.7)	1 (1.9)	3 (8.8)
Ethnicity, n (%)			
Hispanic or Latino	5 (5.8)	2 (3.8)	3 (8.8)
Caucasian	67 (77.9)	42 (80.8)	25 (73.5)
Unknown†	14 (16.3)	8 (15.4)	6 (17.6)
JADAS-27 score, mean (SD)	15.1 (7.1)	14.8 (6.7)	15.5 (7.8)
Physician global assessment of disease activity (VAS 0–100 mm), mean (SD)	47.3 (21.1)	47.2 (20.2)	47.4 (22.9)
Parent/patient global assessment of overall well-being (VAS 0–100 mm), mean (SD)	48.5 (28.3)	50.0 (27.6)	46.3 (29.5)
Childhood Health Assessment Questionnaire–Disability Index, mean (SD)	0.8 (0.6)	0.8 (0.6)	0.8 (0.7)
Number of joints with active arthritis, mean (SD)	7.7 (7.5)	6.2 (3.4)	10.0 (10.6)
Number of joints with limited range of motion, mean (SD)	5.5 (4.7)	4.9 (3.3)	6.6 (6.3)
C-reactive protein standardised value (mg/L), mean (SD)	18.6 (32.0)	24.0 (38.8)	10.4 (14.0)
Total enthesitis count, mean (SD)	2.6 (2.5); n=85	2.7 (2.2); n=52	2.3 (3.0); n=33
Total dactylitis count, mean (SD)	1.0 (2.2); n=82	0.4 (1.4); n=48	1.8 (2.7); n=34
MTX use, n (%) yes	56 (65.1)	33 (63.5)	23 (67.6)

*Asian and other races.

†ethnicity not reported or unknown.

ERA, enthesitis-related arthritis; JADAS, Juvenile Arthritis Disease Activity Score; JIA, juvenile idiopathic arthritis; JPsA, juvenile psoriatic arthritis; MTX, methotrexate; TP, treatment period; VAS, visual analogue scale.

Overall, 75 patients (ERA/JPsA, 44/31) entered TP2 to be randomised 1:1 to continue secukinumab or newly receive placebo, with 67 patients completing TP2 and 26 completing TP3 (online supplemental figure S2). The most common reason for patients to discontinue the study early was lack of efficacy.

### Time to JIA flare (primary endpoint)

The study was completed with last patient’s last visit achieved. A total of 31 JIA flares had occurred, and all 67 remaining patients had reached week 104 (in TP2/TP3). In TP2, flare events occurred in 10/37 (27%) in the secukinumab group versus 21/38 (55%) in the placebo group. The median time to flare was not reached for the secukinumab group and was 453 (95% CI 114 to not calculable) days for the placebo group. The study met its primary end point and demonstrated a statistically significant prolongation in time to disease flare in TP2 for the combined JIA categories of ERA and JPsA in the secukinumab group compared with the placebo group (HR, 0.28 (95% CI 0.13 to 0.63), p<0.001) (figure 1A). The estimated mean time to disease flare in the placebo group was 453 days and could not be formally calculated for the secukinumab group as fewer than 50% of patients in this group had flared at the time of study completion. Based on Kaplan-Meier estimation, the probability of remaining free of disease flares after 1 year was 76.7% (95% CI 58.7 to 87.6) for the secukinumab group versus 54.3% (95% CI 37.1 to 68.7) for the placebo group (figure 1A). Subgroup analysis by JIA category also showed that time to disease flare was longer in secukinumab treatment versus placebo for ERA (HR, 0.45 (95% CI 0.16 to 1.28)) (figure 1B) and JPsA (HR, 0.15 (95% CI 0.04 to 0.57)) (figure 1C).

### Key secondary end points

Onset of JIA ACR30 response occurred as early as week 1 (33.7%) and increased to 87.2% (75/86) at week 12. A total of 67.4% (58/86) of patients achieved a JIA ACR70 response while 34.9% (30/86) achieved JIA ACR-ID status at week 12 (non-responder imputation analysis) (figure 2A). During TP2, compared with patients randomised to placebo, a higher proportion of patients receiving secukinumab achieved JIA ACR30/50/70/90/100 response and JIA ACR-ID status at the end of TP2, which refers to each patient’s last assessment in TP2 (figure 2B). Rates of JIA patients who reported inactive disease in the secukinumab group were higher than placebo group (47.2% vs 37.8%).

**Figure 3:**
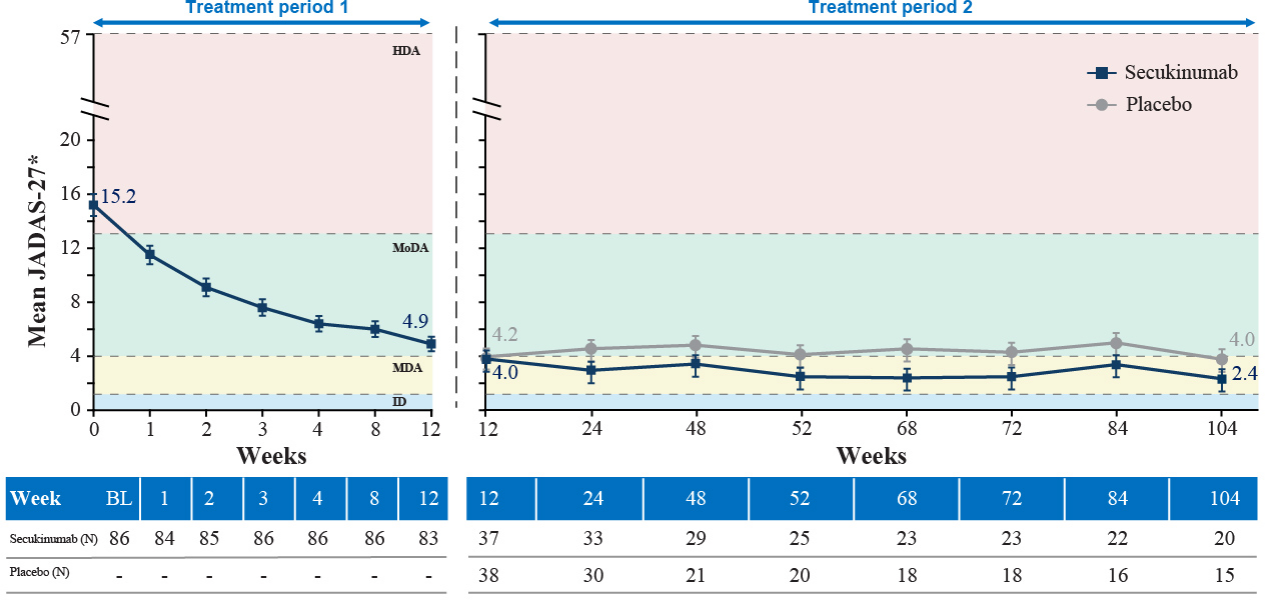
Mean JADAS-27 in TP1 and TP2 in the overall JIA population Full analysis set. MMRM analysis. ^*^Least square mean values. JADAS-27 ranges from 0 to 57 (higher scores indicate more disease activity). HDA, high disease activity; ID, inactive disease; JADAS-27, Juvenile Arthritis Disease Activity Score in 27 joints; JIA, juvenile idiopathic arthritis; MDA, minimal disease activity; MMRM, mixed-effect model repeated measure; MoDA, moderate disease activity; N, total number of patients in the treatment group; TP, treatment period

In the overall JIA population, with OL secukinumab, a notable reduction of mean JADAS-27 was observed up to week 12 reaching moderate disease activity and reached minimal disease activity in both secukinumab and placebo groups in TP2 (figure 3). In ERA and JPsA categories, JADAS-27 reached moderate and minimal disease activity, respectively, with OL secukinumab in TP1. In TP2, the scores reached minimal disease activity with secukinumab treatment in ERA and inactive disease in JPsA, whereas with placebo, the scores reached moderate and minimal disease activity in ERA and JPsA categories, respectively (online supplemental figure S3).

### Safety

Safety data were available for a total of 141.5 PY over the entire treatment period (secukinumab, 71.3 PY; placebo in TP2, 70.2 PY). During the study, 79 patients (91.1%) reported at least one AE in the entire TP (table 2). Eight patients (9.3%) discontinued the study due to AEs (secukinumab, 3 (6.3%); placebo, 5 (13.2%)) and 11 (12.8%) patients reported SAEs). The most frequent TEAEs were nasopharyngitis (27 (31.4%)), nausea (19 (22.1%)), upper respiratory tract infection (19 (22.1%)) and diarrhoea (17 (19.8%)).

**Table 2:** Safety summary for the entire treatment period (safety set)

Safety events	TP1	TP2		Entire secukinumab exposure period
	Secukinumab, N=86	Secukinumab, N=37	Placebo, N=38	Total, N=86
AEs, n (%) (PT)	56 (65.1)	34 (91.9)	29 (76.3)	79 (91.9)
SAE, n (%) (PT)	2 (2.3)	5 (13.5)	0	11 (12.8)
AEs leading to study discontinuation	1 (1.2)	2 (5.4)	5 (13.2)	8 (9.3)
Death	0	0	0	0
Most frequent TEAEs, n (%) (PT)				
Nasopharyngitis	5 (5.8)	14 (37.8)	6 (15.8)	27 (31.4)
Nausea	6 (7.0)	7 (18.9)	3 (7.9)	19 (22.1)
Upper respiratory tract infection	6 (7.0)	6 (16.2)	6 (15.8)	19 (22.1)
Diarrhoea	1 (1.2)	9 (24.3)	2 (5.3)	17 (19.8)
Cough	1 (1.2)	7 (18.9)	4 (10.5)	13 (15.1)
Arthralgia	2 (2.3)	6 (16.2)	3 (7.9)	12 (14.0)
Oropharyngeal pain	5 (5.8)	4 (10.8)	2 (5.3)	12 (14.0)
Headache	5 (5.8)	3 (8.1)	3 (7.9)	12 (14.0)
Fever	2 (2.3)	6 (16.2)	2 (5.3)	12 (14.0)

Secukinumab and placebo in TP2 groups have not been compared as the study design and the exposure times for these groups were different over the entire TP. In addition, it cannot be ruled-out that events occurring in TP2 under placebo are due to a spill-over effect by the previous secukinumab treatment in TP1.

AE, adverse event; IR, incidence rate per 100 subject years; PT, preferred term; SAE, serious adverse event; SOC, primary system organ class; TEAEs, treatment emergent AEs; TP, treatment period.

One patient with ERA had a medical history of uveitis that was non-recurring during the study. Two ERA patients, both aged 16 years, reported AE of acute anterior uveitis of mild or moderate severity that was not considered related to the study drug by the investigators and resolved with topical therapy; hence, the dose of study drug remains unchanged, and both the patients recovered and completed the study. One JPsA patient (2.7%) in the secukinumab group newly reported Crohn’s disease during the study. There was no family history of IBD. The patient experienced disease flare and discontinued the study drug due to Crohn’s disease on Day 127, entered post-treatment follow-up period and completed the same. The investigator suspected a causal relationship to secukinumab/placebo. There were no cases of mycobacterial infections, hepatitis B reactivation, malignancy or deaths. Only one patient reported injection-site reaction. No treatment-emergent antidrug antibodies were detected in any sample of patients treated with secukinumab during the study.

## Discussion

Secukinumab is a monoclonal antibody targeting IL-17A that was found to result in rapid improvement of arthritis, dactylitis and enthesitis in children with ERA and JPsA. This study met its primary end point and demonstrated that time to disease flare in TP2 was significantly longer with secukinumab treatment than placebo in the overall JIA population. At the tested weight-stratified dosages, secukinumab markedly decreased the flare risk by 72% compared with placebo, and there was no new safety signal.

There remains a high unmet medical need for therapies indicated for ERA and JPsA as there is a dearth of tested therapies.^18^ In order to limit or even avoid the negative impact of ERA and JPsA on patient development, QoL or disease-associated joint damage, rapid and sustained control of disease signs and symptoms is recommended,^19 20^ which can be achieved with early initiation of anti-inflammatory treatment in JIA.^21^ Current treatment guidelines recommend initial bDMARD treatment for patients with risk factors, high disease activity and those who are intolerant to csDMARDs.^21 22^

We consider secukinumab an important new treatment option for children with ERA and JPsA as secukinumab resulted in a rapid and profound improvement of signs and symptoms of both ERA and JPsA in this study, including the resolution of dactylitis and enthesitis. The achievement of inactive disease, a preferred treatment target for JIA, was achieved in over 30% of patients by week 12, and in over 40% of patients who received secukinumab throughout the entire study. Resolution of enthesitis is highly impactful given the known profound detrimental effects of enthesitis, including pain, in both adults and children.^23^

The responses in children with JPsA receiving secukinumab in this study are consistent with the findings from studies in patients with PsA where secukinumab has demonstrated sustained improvements in ACR responses, in resolution of enthesitis and dactylitis, in skin clearance with sustained improvement across the six key manifestations of PsA through 5 years.^24–26^ ERA is considered the paediatric counterpart of nr-axSpA^2^ and children with ERA also experienced a profound improvement of signs and symptoms of their disease. The role of IL-17A in spondyloarthritide manifestations of skin, joints and entheses is well known, and profound improvement with secukinumab was reported in patients with PsO, PsA and AS.^11 27^

Prior bDMARD exposure was an exclusion criterion for study participation. Earlier studies in JIA support that exposure to prior bDMARDs decreases the response rate to subsequent treatments in medication trials.^28 29^ Although this trial does not provide information about secukinumab efficacy in the setting of prior bDMARD exposure, we note that a retrospective study in patients with ERA who failed anti-TNF treatment reported significant improvement in JIA disease activity as measured by JADAS with secukinumab treatment.^30^ Consistent with this finding, patients in this study experienced a rapid, profound, and sustained decrease in disease activity as measured by JADAS-27 for up to 2 years.

There was only one reported injection-site reaction during the study, and no antisecukinumab antibodies were detected. Indeed, secukinumab was generally well tolerated, and its safety profile in this study population of patients with ERA or JPsA was consistent with that observed in adult patients with axial spondyloarthritis and PsA.^8 9 25^

There was one reported case of Crohn’s disease that was categorised as an important potential risk, consistent with prior reports in the medical literature based on a postmarketing study in Vigibase.^31^ Anterior chronic non-infectious uveitis is quite common, especially with early onset of JPsA,^32^ but was not observed in this study. On the contrary, two cases of new acute anterior uveitis were reported, both regarded unrelated to the study treatment by the treating physicians, and a patient with a history of acute anterior uveitis prior to baseline did not experience reactivation of uveitis during the study.

Limitations of the trial must be considered. Secukinumab efficacy was assessed indirectly by the occurrence of JIA flares. Owing to the large number of placebo-treated patients who met flare criteria in TP2 and who stopped placebo when entering TP3, observed differences in efficacy between secukinumab versus placebo may be blunted. The trial population was relatively small and predominantly white but was in line with other JIA trials. Thus, it was not possible to detect rare AEs. Another limitation of this study is the lack of data on skin manifestations, especially in JPsA patients, although it has been acknowledged that secukinumab demonstrated sustained efficacy across various skin outcomes in previously reported randomised trials in paediatric patients with PsO.^33^

In conclusion, secukinumab demonstrated efficacy and safety in the JIA categories of ERA and JPsA. The significantly longer time to disease flare in TP2 and improvement in disease activity observed establish secukinumab as a candidate in the treatment of patients with ERA and JPsA.

## Data Availability

Data are available upon reasonable request. The datasets generated and analysed for this study are not publicly available. Novartis will review requests for data from qualified external researchers for scientific merit. All patient-level data must obscure patient identity, to respect patient privacy and conform to applicable laws and regulations. Any requests should be made to Luminita Pricop, Novartis Pharmaceutical Corporation, at luminita.pricop@novartis.com.
