# Supplementary material for: Secukinumab in enthesitis-related arthritis and juvenile psoriatic arthritis: a randomised, double-blind, placebo-controlled, treatment withdrawal, phase 3 trial
Source: Ann Rheum Dis. 2022 Aug 12;82(1):154–60. doi: 10.1136/ard-2022-222849 (PMC9811076; doi:10.1136/ard-2022-222849)
Supplement: Supplementary data [file ard-2022-222849supp001.pdf]

Secukinumab in Enthesitis-Related Arthritis and Juvenile Psoriatic Arthritis: a randomised, double-blind, placebo-controlled, treatment withdrawal, phase 3 trial

Hermine I Brunner*, Ivan Foeldvari, Ekaterina Alexeeva, Nuray A Ayaz, Inmaculada C Penades, Ozgur Kasapcopur, Vyacheslav G Chasnyk, Markus Hufnagel, Zbigniew Zuber, Grant S Schulert, Seza Ozen, Adelina Rakhimyanova, Athimalaipet V Ramanan, Christiaan Scott, Betul Sozeri, Elena Zholobova, Ruvie Martin, Xuan Zhu, Sarah Whelan, Luminita Pricop, Alberto Martini, Daniel J Lovell and Nicolino Ruperto, on behalf of Paediatric Rheumatology INternational Trials Organization (PRINTO) and Pediatric Rheumatology Collaborative Study Group (PRCSG)

Corresponding author:

Prof. Hermine I Brunner,
UC Department of Pediatrics,
Cincinnati Children's Hospital,
University of Cincinnati, Cincinnati, OH 45229, USA
3333 Burnet Avenue, MC 4010
Email: Hermine.Brunner@cchmc.org
Phone: +1 513-636-7982

Brunner HI, *et al. Ann Rheum Dis* 2022; 82:154–160. doi: 10.1136/ard-2022-222849

Supplementary Appendix

Inclusion and exclusion criteria

Inclusion criteria

1. Male or female patients in the age range of ≥2 years to <18 years at screening
2. Confirmed diagnosis of enthesitis-related arthritis (ERA) according to the International League of Associations for Rheumatology (ILAR) classification criteria or juvenile psoriatic arthritis (JPsA) according to the modified ILAR criteria that had occurred at least 6 months prior to screening
3. Active disease defined as:
   - ≥3 active joints (swollen or if not swollen must be tender with limited range of motion) at baseline and
   - ≥1 site of active enthesitis at baseline or documented history
4. Inadequate response to ≥1
   - nonsteroidal anti-inflammatory drugs (NSAIDs) for ≥1 month or
   - disease modifying anti-rheumatic drugs (DMARDs) for ≥2 months
5. No concomitant use of second line disease-modifying and/or immunosuppressive drugs except the stable doses of
   - methotrexate (maximum of 20 mg/m^2 body surface area/week) for at least 4 weeks prior to baseline visit, and folic/folinic acid supplementation
   - sulfasalazine (ERA patients only) <50 mg/kg/day with a maximum of 3000 mg/day for at least 4 weeks prior to baseline visit

- oral corticosteroid at a prednisone equivalent dose of <0.2 mg/kg/day or up to 10 mg/day maximum, whichever was less, for at least 7 days prior to baseline
- not more than one NSAID for at least 1 week prior to baseline

6. Negative results for
   - QuantiFERON test or
   - purified protein derivative (PPD) test if required by local guidelines or if the patient was <5 years of age
     - positive PPD was defined as >15 mm induration in children >4 years and >10 mm for children <4 years
     - patients with positive PPD results participated in the study if further work up established that the patient had no evidence of active tuberculosis (TB). In presence of latent TB, treatment was initiated according to local country guidelines for a minimum of 4 weeks before baseline
7. Signed and written informed consent from parent/legal guardian of the child prior to any study related activity or assessment

Exclusion criteria

1. Use of other investigational drugs within 4 weeks or 5 half-lives of baseline, or until the expected pharmacodynamic effect had returned to baseline, whichever was longer
2. History of hypersensitivity to any of the study drugs or its excipients or to drugs of similar chemical classes
3. Patients with active uncontrolled inflammatory bowel disease or uveitis
4. Patients who had received biologic immunomodulating agents, including but not limited to tumor necrosis factor (TNF)α inhibitors, T-cell costimulatory, anti-IL6, anti-

IL1, cell-depleting therapies (alemtuzumab, anti-CD4, anti-CD5, anti-CD3, and anti-CD19), secukinumab or other biologic drugs directly targeting interleukin (IL)-17 or IL-17 receptor or any investigational immunomodulating agent

5. Patients taking any non-biologic DMARD except for methotrexate or sulfasalazine (for ERA patients)
6. Patients fulfilling any ILAR diagnostic juvenile idiopathic arthritis (JIA) category other than ERA or JPsA
7. Patients taking medications prohibited by the protocol
8. Patients taking high potency opioid analgesics (morphine equianalgesic or higher) including but not limited to methadone, hydromorphone and morphine
9. Any intramuscular/intravenous/intra-articular corticosteroid treatment within 4 weeks before baseline
10. Patients with active or recurrent bacterial, fungal or viral infection including known infection with HIV, Hepatitis B, and Hepatitis C at baseline
11. Patients with active TB or latent TB who are unwilling or unable to complete a minimum of 4 weeks of latent TB treatment before initiating treatment with secukinumab
12. History or current diagnosis of ECG abnormalities indicating significant risk of safety for patients participating in the study such as:
    - concomitant clinically significant cardiac arrhythmias, e.g., sustained ventricular tachycardia, and clinically significant second or third degree AV block without a pacemaker

- history of familial long QT syndrome or known family history of Torsades de Pointes

13. Pregnant or lactating females (pregnancy defined as the state of a female after conception and until the termination of gestation, confirmed by a positive human chorionic gonadotropin laboratory test)
14. Female patients (<18 years of age) of childbearing potential (menarchal or becoming menarchal during the study) who did not agree to abstinence or, if sexually active, did not agree to the use of contraception
15. Active ongoing inflammatory diseases other than JPsA/ERA that might confound the benefit of secukinumab therapy
16. Underlying metabolic, hematologic, renal, hepatic, pulmonary, neurologic, endocrine, cardiac, infectious or gastrointestinal conditions which immunocompromises the patient and/or places the patient at unacceptable risk for participation in a study
17. Significant medical problems or diseases, including but not limited to uncontrolled hypertension and uncontrolled diabetes
18. History of clinically significant liver disease or liver injury as indicated by abnormal liver function tests such as alanine aminotransferase, aspartate aminotransferase, alkaline phosphatase, or serum bilirubin. The criteria for diagnosis included:
    - any single parameter not exceeding 2× upper limit of normal (ULN). A single parameter elevated up to 2× ULN were re-checked again prior to baseline, to rule out lab error

- if the total bilirubin concentration increased above 2× ULN, it was differentiated into the direct and indirect reacting bilirubin; with serum bilirubin not exceeding 1.6 mg/dL (27 μmol/L) in either case

19. Patients with total WBC count <3000/μL, or platelets <100000/μL or neutrophils <1500/μL or hemoglobin <8.5 g/dL (85 g/L) at screening
20. History of lymphoproliferative disease or any known malignancy within the past 5 years (except for treated basal cell carcinoma or actinic keratoses with no evidence of recurrence in the past 3 months and carcinoma in situ of the cervix or non-invasive malignant colon polyps that had been removed)
21. Current severe progressive or uncontrolled disease rendering the patient unsuitable for the trial
22. Inability or unwillingness to undergo repeated venipuncture (e.g., because of poor tolerability or lack of access to veins)
23. Any medical or psychiatric condition that would preclude the participant from adhering to the protocol or completing the study per protocol
24. History or evidence of ongoing alcohol or drug abuse, within the last 6 months before baseline
25. Plans for administration of live vaccines during the study period or within 6 weeks preceding baseline

Further details on patient randomisation and masking

Patients eligible to enter treatment period (TP)2 were randomised 1:1 to continue secukinumab or newly start placebo. Allocation of patients to the treatment arms was performed using an Interactive Response Technology (IRT) system. TP2 was blinded to the parents/patients,

investigators, and sponsor. Randomisation in TP2 was stratified by JIA category (ERA, JPsA). The site personnel (study coordinator or specified designee) were required to enter or select information including but not limited to the user's identification and password, protocol number, patient number, and patient's date of birth. The site personnel were then provided with a treatment assignment and dispensable unit or container number when drug was being supplied via the IRT system.

Additional details on drug administration and procedures

Secukinumab was administered subcutaneously in pre-filled syringes of 75 mg for patients with body weight of <50 kg or 150 mg for patients with body weight ≥50 kg. During TP1, five weekly loading doses of secukinumab were administered up to week 4, followed by 4-weekly injections thereafter. Patients could continue stable background therapies with NSAIDs, methotrexate (≤20 mg/m^2/week),[1] sulfasalazine (ERA patients only; ≤50 mg/kg/day or 3 g/day, whichever was lower), and oral glucocorticoids (≤0.2 mg/kg/day of prednisone equivalent or ≤10 mg/day, whichever was lower). Intra-articular glucocorticoid injections were not permitted within 4 weeks prior to baseline, during TP1 or TP2; but patients were allowed to receive injections in up to four joints per 24-week period during TP3.

Study endpoints, outcomes and assessments

Primary endpoint

- Time to disease flare during TP2

Secondary endpoints

- Efficacy of secukinumab treatment for all subjects and each JIA category in TP1 measured using:

  - JIA American College of Rheumatology (ACR)30/50/70/90/100
  - Inactive disease status
  - JIA ACR core components
  - Juvenile Arthritis Disease Activity Score (JADAS)
  - Total enthesitis count
  - Total dactylitis count
- Withdrawal effect of secukinumab treatment for all subjects and each JIA category in TP2 assessed by:
  - JIA ACR30/50/70/90/100
  - Inactive disease status
- Secukinumab serum concentrations and derived pharmacokinetic parameters in TP1
- Safety/tolerability:
  - Evaluation of adverse events/serious adverse events
  - Laboratory values
  - Vital signs
- Immunogenicity assessment
  - Anti-drug antibodies

Exploratory endpoints

- Time to disease flare for ERA and JPsA in TP2
- Withdrawal effect of secukinumab treatment for all subjects and each JIA category in TP2 assessed by:
  - JIA ACR core components
  - JADAS

  - Total enthesitis count
  - Total dactylitis count
  - Swollen joint count
  - Tender joint count
  - Overall back pain (using visual analog scale [VAS]) in ERA subset only
  - Nocturnal back pain (using VAS) in ERA subset only
  - Psoriasis (PsO) global assessment (Investigator's Global Assessment [2011 modified version (IGA (Mod 2011)])[2] in JPsA subset only
  - Percentage of total body surface area affected by PsO
  - Modified Schober's test
- Juvenile Spondyloarthritis Disease Activity Index (JSpADA) at weeks 12, 52, 104 and at time of disease flare in TP2
- Correlations of JSpADA[3] with JADAS, patient global assessment of disease activity (VAS) and the Child Health Assessment Questionnaire (CHAQ) during entire study period
- Efficacy of secukinumab in TP3 after experiencing disease flare in TP2 measured by:
  - JIA ACR 30/50/70/90/100
  - Inactive disease status
  - JIA ACR core components
- Efficacy of secukinumab in entire study period measured by:
  - JIA ACR30/50/70/90/100
  - Inactive disease
  - Proportion of patients achieving clinical remission

- Potential proteomic biomarkers associated with treatment response to secukinumab in entire study period

Assessment of JIA ACR core set variables and other efficacy assessments

The six validated JIA ACR core set variables that were assessed are as follows:[4] physician's global evaluation of overall disease activity (VAS 0–100 mm); patient/parent assessment of overall well-being (VAS 0–100 mm); number of joints with active arthritis, i.e. with swelling or in the absence of swelling, limitation in range of motion accompanied by either joint tenderness or pain on motion; number of joints with limitation of motion; cross-culturally adapted and validated version of Childhood Health Assessment Questionnaire-Disability Index (CHAQ-DI)[5] as a measure of physical ability; and C-reactive protein (CRP) as a measure of systemic inflammation.

The following efficacy assessments were assessed and confirmed in real-time, according to validated criteria, by independent evaluators at the PRINTO/PRCSG centralized coordinating centers: JIA core set variables by certified joint assessors; JIA ACR30/50/70/90/100 response; JIA flare; JIA ACR-ID and JADAS-27 CRP scores and disease status. The JADAS-27 cutoffs published in 2021 were used: high (>13.0), moderate (4.1–13.0), minimal (1.5–4.0) and inactive disease (JADAS-ID) (≤1.4).[6]

Additional results of other key endpoints

At the end of TP1 and TP2, all JIA ACR core set variables improved from baseline, including >50% improvement in patient-reported outcomes (CHAQ-DI, parent/patient-reported assessment of overall well-being) (Fig. S4 and Table S2).

In patients with enthesitis or dactylitis at baseline (N=71 or N=22, respectively), resolution of enthesitis or dactylitis was reported in 69.0% and 59.1% patients, respectively, using non-

responder imputation analysis for missing data (Fig. S5). Among 31 patients with enthesitis and 12 patients with dactylitis at baseline who entered TP2 and were randomised to receive secukinumab during TP2, 45.2% and 25% of patients, respectively, showed sustained resolution of enthesitis and dactylitis for six or more months. Similar results were observed in patients with ERA and JPsA (Table S2).

Additional results on safety

For the entire study (N=86), the exposure-adjusted incidence rates (IRs) (95% CI) for AEs per 100 patient-years (PY) of follow-up was 290.7 (230.2 to 362.3). The IR was 355.4 (258.2 to 477.1) for secukinumab patients who did not take any placebo throughout the study (N=48), and 236.6 (164.8 to 329.1) for all patients who took placebo in TP2 and secukinumab in other TPs (N=38). Similarly, the IR for SAEs with secukinumab in the entire study was 8.2 (4.1 to 14.6), whereas it was 10.4 (4.2 to 21.4) with patients who received secukinumab throughout the study and 5.9 (1.6 to 15.2) in all patients who took placebo in TP2 and secukinumab in other TPs. The IR (95% CI) per 100 PY of follow-up for the *Medical Dictionary for Regulatory Activities* (MedDRA) System Organ Class ‘Infections and infestations’ was numerically higher with secukinumab than placebo (134.2 [104.2 to 170.1] vs. 112.5 [75.9 to 160.7]). The same was true for ‘Respiratory, thoracic, and mediastinal disorders’ (IR [95% CI] secukinumab versus placebo: 23.1 [14.9 to 34.0] vs. 15.4 [7.1 to 29.3]).

Figure S1. Study design

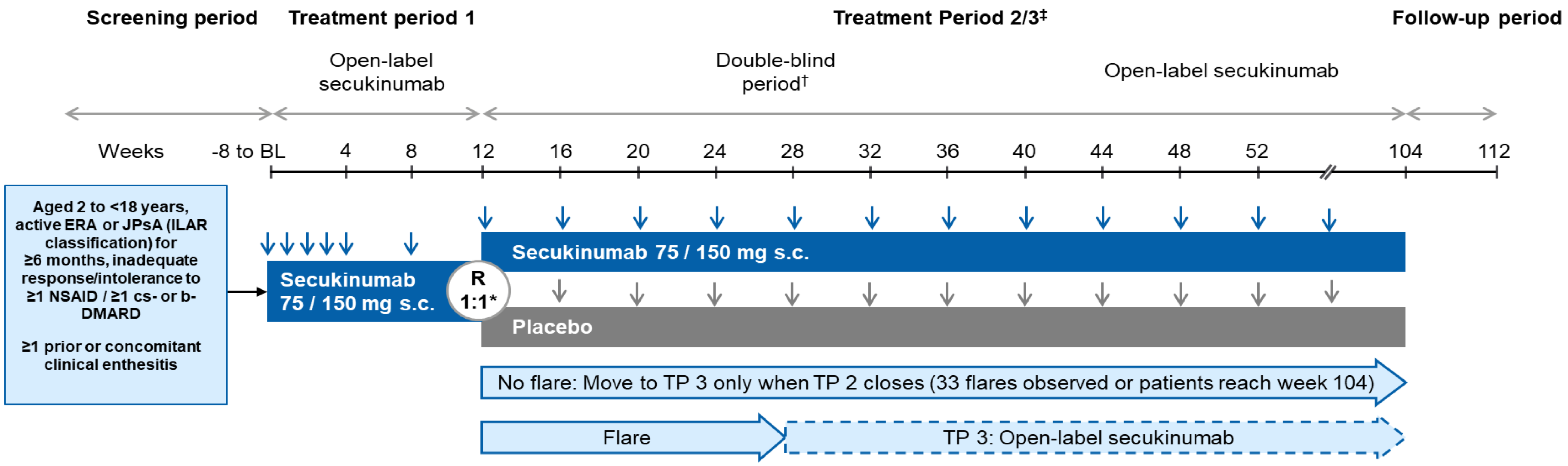

*Patients only randomised if they achieve ACR 30. If ACR30 is not achieved, patient is discontinued from the study. †Closes when 33 patients experience a disease flare. ‡Treatment period 2 is a withdrawal-period

BL, baseline; cs- or b-DMARD, conventional synthetic or biological disease-modifying antirheumatic drug; ERA, enthesitis-related arthritis; ILAR, International League Against Rheumatism; JPsA, juvenile psoriatic arthritis; NSAID, non-steroidal anti-inflammatory drug; R, randomised; s.c. subcutaneous; TP, treatment period

Brunner HI, *et al. Ann Rheum Dis* 2022; 82:154–160. doi: 10.1136/ard-2022-222849

Figure S2. Patient disposition

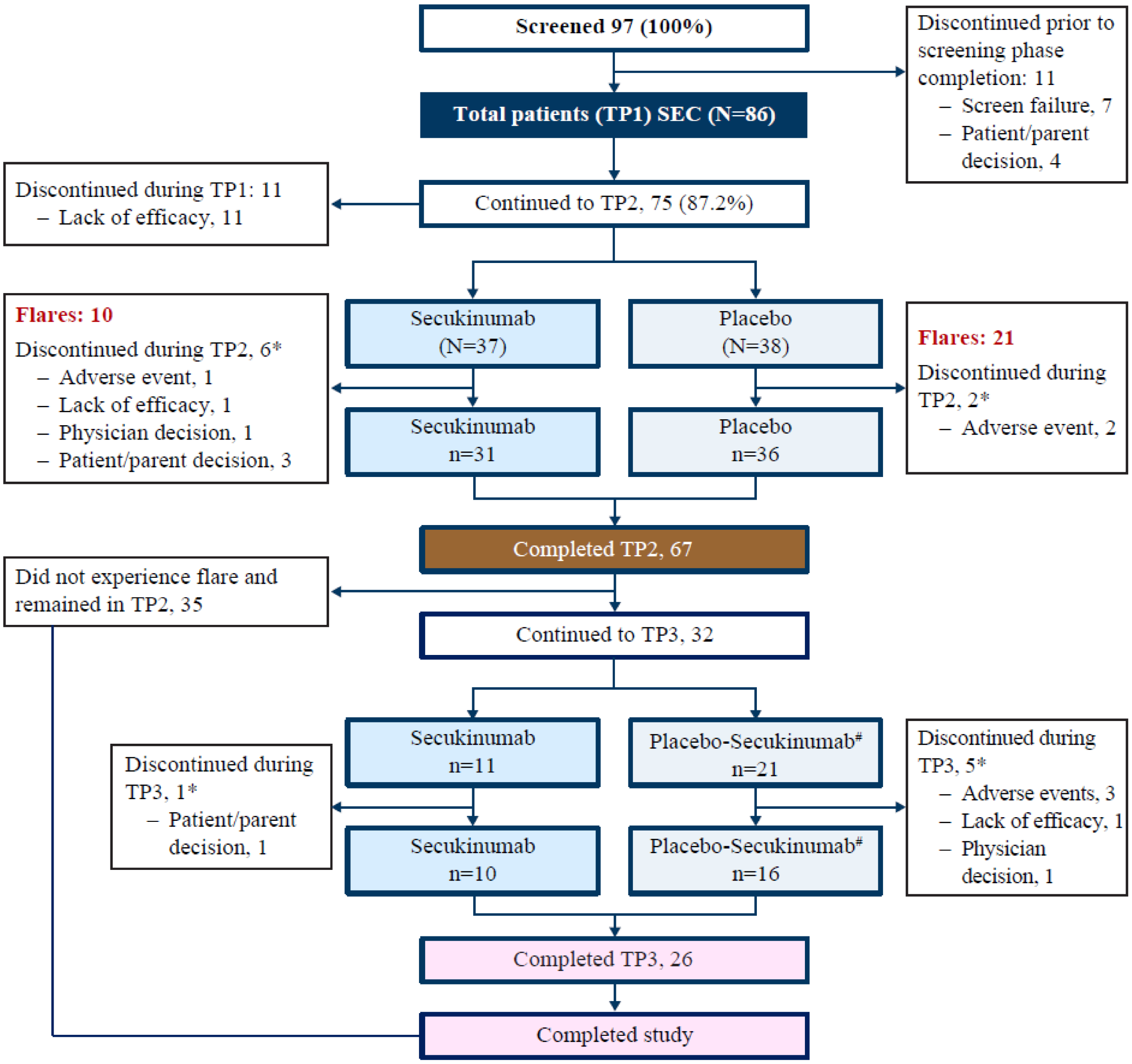

*Includes patients who completed the given period and discontinued prematurely from the study treatment on the same date; #Patients in the placebo group in TP2 switched to open-label secukinumab in TP3.

SEC, secukinumab; TP, treatment period

Brunner HI, *et al. Ann Rheum Dis* 2022; 82:154–160. doi: 10.1136/ard-2022-222849

Figure S3. Improvement in mean JADAS-27 in (A) ERA and (B) JPsA subcategories in TP1 and TP2

A)

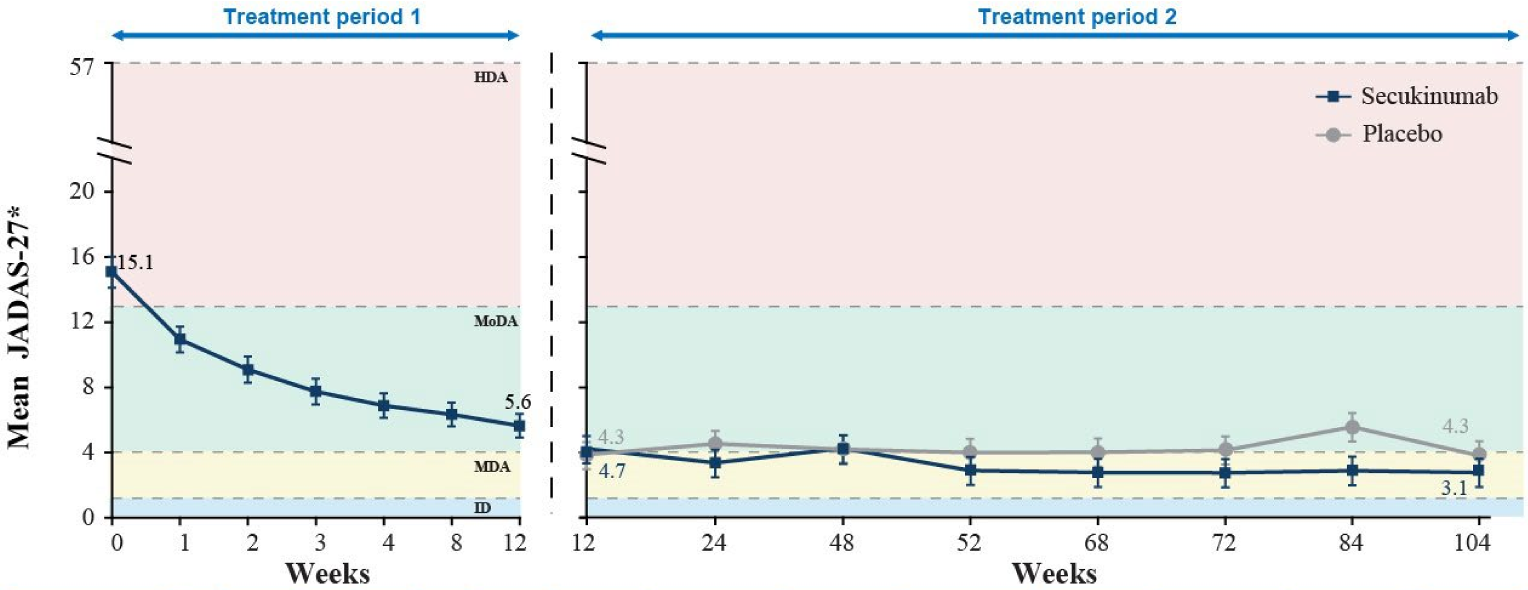

Week	BL	1	2	3	4	8	12	12	24	48	52	68	72	84	104
Secukinumab (N)	52	50	51	52	52	52	51	22	20	17	15	14	14	13	13
Placebo (N)	-	-	-	-	-	-	-	22	20	13	13	13	13	12	11

Full analysis set. MMRM analysis.
*Least square mean values. JADAS-27 ranges from 0 to 57 (higher scores indicate more disease activity).
ERA, enthesitis-related arthritis; HDA, high disease activity; ID, inactive disease; JADAS-27, Juvenile Arthritis Disease Activity Score in 27 joints; MDA, minimal disease activity; MMRM, mixed-effect model repeated measure; MoDA, moderate disease activity; N, total number of patients in the treatment group; TP, treatment period

B)

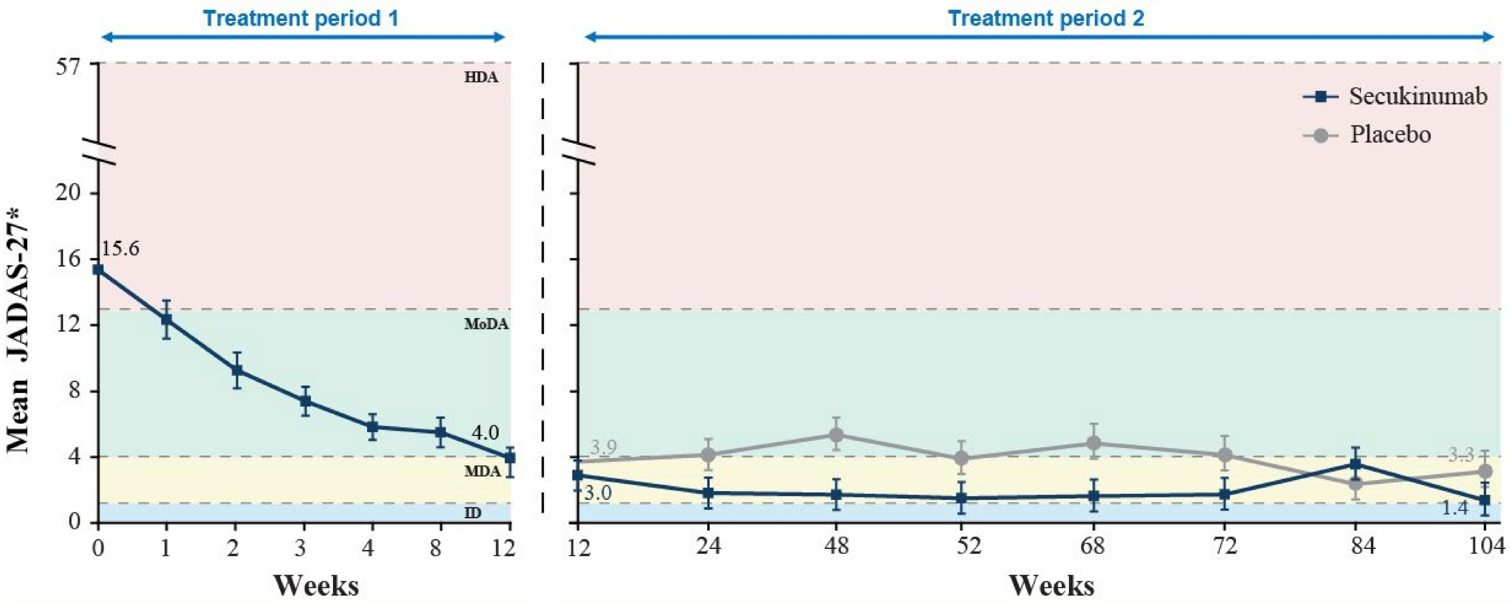

Week	BL	1	2	3	4	8	12	12	24	48	52	68	72	84	104
Secukinumab (N)	34	34	34	34	34	34	32	15	13	12	10	9	9	9	7
Placebo (N)	-	-	-	-	-	-	-	16	10	8	7	5	5	4	4

Full analysis set. MMRM analysis.

*Least square mean values. JADAS-27 ranges from 0 to 57 (higher scores indicate more disease activity).

HDA, high disease activity; ID, inactive disease; JADAS-27, Juvenile Arthritis Disease Activity Score in 27 joints; JPsA, juvenile psoriatic arthritis; MDA, minimal disease activity; MMRM, mixed-effect model repeated measure; MoDA, moderate disease activity; N, total number of patients in the treatment group; TP, treatment period

Figure S4. Improvement of JIA ACR core set variables from baseline to the end of TP1

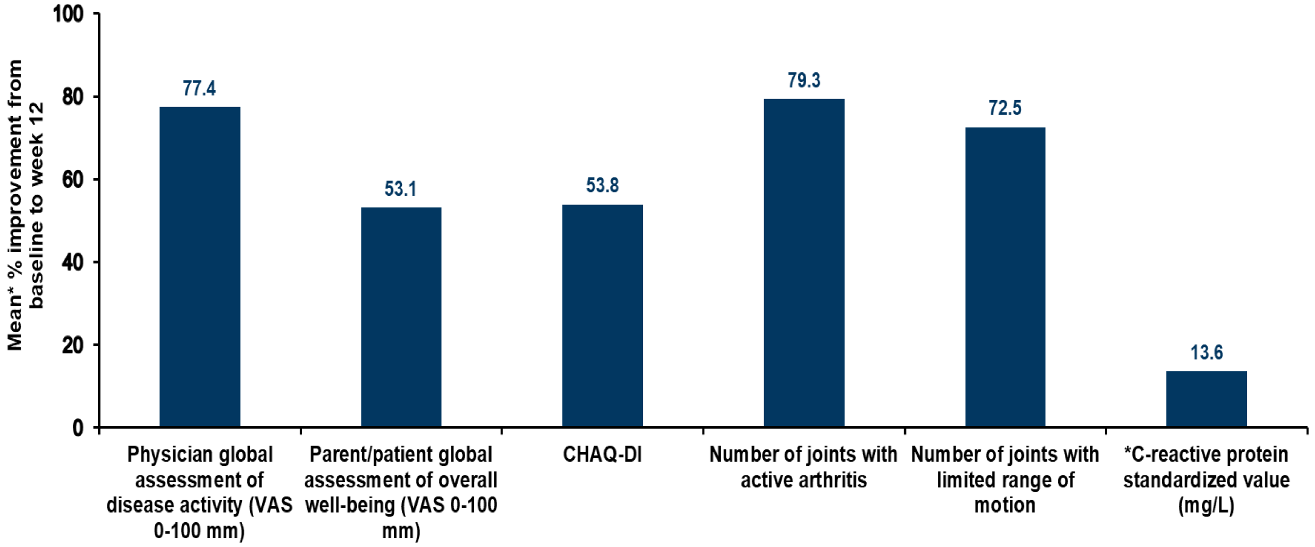

*C-reactive protein is shown as median percent improvement from baseline, due to outliers of C-reactive protein values. Baseline is defined as the last observation on the day of or before the first dose of study drug. Only patients with a non-zero value at baseline and a value at week 12 are included.

ACR, American College of Rheumatology; CHAQ-DI, Childhood Health Assessment Questionnaire-Disability Index; JIA, juvenile idiopathic arthritis; VAS, visual analog scale

Brunner HI, *et al. Ann Rheum Dis* 2022; 82:154–160. doi: 10.1136/ard-2022-222849

Figure S5. Resolution of enthesitis and dactylitis in the overall JIA population

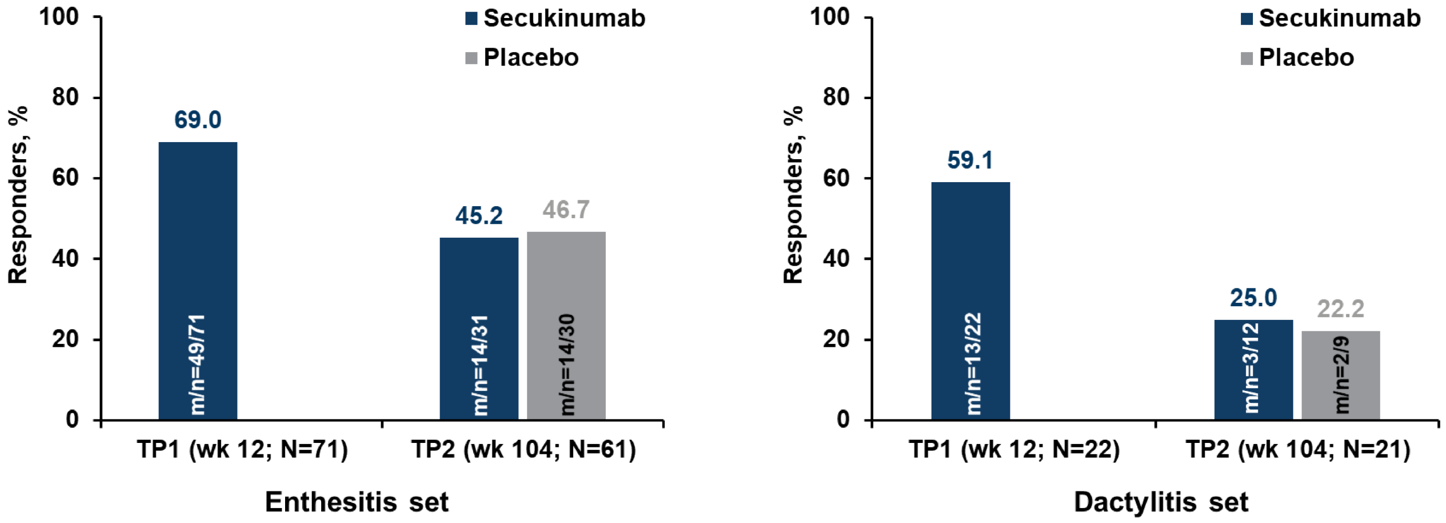

NRI analysis. FAS consisted of all patients who received at least one dose of study drug.

Enthesitis/dactylitis set included all FAS patients who had enthesitis/dactylitis at baseline.

FAS, full analysis set; JIA, juvenile idiopathic arthritis; m, number of patients with a response; N, total number of patients with enthesitis/dactylitis; NRI, non-responder imputation; n, number of patients with enthesitis/dactylitis in each group; TP, treatment period; wk, week

Table S1. Baseline demographics and clinical characteristics of all JIA patients randomised into TP2 and each JIA category

Characteristic	ERA (N=44)		JPsA (N=31)		JIA (N=75)	
	SEC (N=22)	PBO (N=22)	SEC (N=15)	PBO (N=16)	SEC (N=37)	PBO (N=38)
**Age (years), mean (SD)**	14.0 (2.5)	13.0 (2.9)	13.1 (3.1)	10.6 (3.7)	13.6 (2.7)	12.0 (3.5)
**Male, n (%)**	18 (81.8)	18 (81.8)	6 (40.0)	9 (56.3)	24 (64.9)	27 (71.1)
**Race, n (%)**						
**White**	22 (100)	21 (95.5)	14 (93.3)	14 (87.5)	36 (97.3)	35 (92.1)
**Other***	0	1 (4.5)	1 (6.7)	2 (12.5)	1 (2.7)	3 (7.9)
**Ethnicity, n (%)**						
**Hispanic or Latino**	0	1 (4.5)	3 (20.0)	0	3 (8.1)	1 (2.6)
**Caucasian**	17 (77.3)	20 (90.9)	8 (53.3)	15 (93.8)	25 (67.6)	35 (92.1)
**Unknown#**	5 (22.7)	1 (4.5)	4 (26.7)	1 (6.3)	9 (24.3)	2 (5.3)
**JADAS-27 score, mean (SD)**	15.9 (8.1)	14.8 (5.7)	14.3 (8.2)	16.7 (8.3)	15.2 (8.0)	15.6 (6.9)
**Physician global assessment of disease activity (VAS 0–100 mm), mean (SD)**	47.3 (22.6)	43.5 (15.6)	40.9 (21.2)	50.3 (24.4)	44.7 (21.9)	46.4 (19.8)
**Parent/patient global assessment of overall well-**	53.2 (29.7)	49.7 (27.9)	37.1 (29.5)	54.6 (28.6)	46.7 (30.3)	51.8 (27.9)

**being (VAS 0–100 mm), mean (SD)**						
**Childhood Health Assessment Questionnaire–Disability Index, mean (SD)**	0.8 (0.6)	0.8 (0.5)	0.5 (0.5)	1.0 (0.8)	0.7 (0.6)	0.9 (0.6)
**Number of joints with active arthritis, mean (SD)**	6.9 (4.7)	5.6 (3.2)	10.4 (9.5)	10.8 (12.5)	8.3 (7.4)	7.8 (8.7)
**Number of joints with limited range of motion, mean (SD)**	5.5 (3.5)	4.7 (2.4)	6.1 (3.2)	7.8 (8.5)	5.8 (3.3)	6.0 (5.9)
**C-reactive protein standardized value (mg/L), mean (SD)**	28.9 (41.2)	26.1 (42.1)	8.1 (9.6)	12.7 (17.8)	20.5 (33.6)	20.5 (34.3)
**Total enthesitis count, mean (SD)**	3.1 (2.6); n=22	2.2 (1.8); n=22	2.1 (3.2); n=15	2.9 (3.0); n=15	2.7 (2.9); n=37	2.5 (2.3); n=37
**Total dactylitis count, mean (SD)**	0.6 (1.7); n=22	0.3 (1.3); n=21	2.0 (2.6); n=15	2.0 (2.9); n=16	1.2 (2.2); n=37	1.0 (2.3); n=37
**MTX use, n (%) Yes**	15 (68.2)	14 (63.6)	11 (73.3)	11 (68.8)	26 (70.3)	25 (65.8)

*Asian and other races; #ethnicity not reported or unknown

ERA, enthesitis-related arthritis; JADAS, Juvenile Arthritis Disease Activity Score; JIA, juvenile idiopathic arthritis; JPsA, juvenile psoriatic arthritis; MTX, methotrexate; N, total number of patients in the treatment group; PBO, placebo; SEC, secukinumab; TP, treatment period; VAS, visual analogue scale

Table S2. Efficacy of secukinumab in ERA and JPsA subcategories at the end of TP1 and TP2

Efficacy Outcomes, %	TP1 (week 12)	TP2	
	SEC	SEC	PBO
**ERA (TP1: N=52; TP2: SEC N=22; PBO N=22)**			
**JIA ACR30**	84.6	90.9	68.2
**JIA ACR50**	78.8	81.8	68.2
**JIA ACR70**	65.4	68.2	54.5
**JIA ACR90**	32.7	45.5	50.0
**JIA ACR100**	26.9	36.4	45.5
**Inactive disease#**	38.5	50.0	50.0
**Resolution of enthesitis***	72.3	72.7	77.3
**Resolution of dactylitis***	50.0	86.4	95.5
**JPsA (TP1: N=34; TP2: SEC N=15; PBO N=16)**			
**JIA ACR30**	91.2	86.7	62.5
**JIA ACR50**	91.2	73.3	56.3
**JIA ACR70**	70.6	66.7	31.3
**JIA ACR90**	47.1	60.0	25.0
**JIA ACR100**	20.6	53.3	25.0
**Inactive disease#**	29.4	46.7	18.8
**Resolution of enthesitis***	62.5	66.7	50.0
**Resolution of dactylitis***	62.5	93.3	62.5

NRI analysis. #Inactive disease: Definition adapted from JIA ACR criteria of Wallace et al., 2011.[7]

*TP1, Enthesitis set (ERA: SEC, N=47; JPsA: SEC, N=24); Dactylitis set (ERA: SEC, N=6; JPsA: N=16).

ACR, American College of Rheumatology; ERA, enthesitis-related arthritis; JIA, juvenile idiopathic arthritis; JPsA, juvenile psoriatic arthritis; N, total number of patients in the treatment group; NRI, non-responder imputation; PBO, placebo; SEC, secukinumab; TP, treatment period

References:

1. Ruperto N, Murray KJ, Gerloni V, *et al*. A randomized trial of parenteral methotrexate comparing an intermediate dose with a higher dose in children with juvenile idiopathic arthritis who failed to respond to standard doses of methotrexate. *Arthritis Rheum* 2004;50(7):2191–201.
2. Langley RG, Feldman SR, Nyirady J, *et al*. The 5-point Investigator's Global Assessment (IGA) Scale: A modified tool for evaluating plaque psoriasis severity in clinical trials. *J Dermatolog Treat* 2015;26(1):23–31.
3. Weiss PF, Colbert RA, Xiao R, *et al*. Development and retrospective validation of the juvenile spondyloarthritis disease activity index. *Arthritis Care Res* 2014;66(12):1775–82.
4. Giannini EH, Ruperto N, Ravelli A, *et al*. Preliminary definition of improvement in juvenile arthritis. *Arthritis Rheum* 1997; 40(7):1202–9.
5. Ruperto N, Ravelli A, Pistorio A, *et al*. Cross-cultural adaptation and psychometric evaluation of the Childhood Health Assessment Questionnaire (CHAQ) and the Child Health Questionnaire (CHQ) in 32 countries. Review of the general methodology. *Clin Exp Rheumatol* 2001;19(4 Suppl 23):S1–9.
6. Trincianti C, Van Dijkhuizen EHP, Alongi A, *et al*. Definition and Validation of the American College of Rheumatology 2021 Juvenile Arthritis Disease Activity Score Cutoffs for Disease Activity States in Juvenile Idiopathic Arthritis. *Arthritis Rheumatol* 2021;73(11):1966–75.
7. Wallace CA, Giannini EH, Huang B, *et al*. American College of Rheumatology provisional criteria for defining clinical inactive disease in select categories of juvenile idiopathic arthritis. *Arthritis Care Res* 2011;63(7):929–36.

Brunner HI, *et al. Ann Rheum Dis* 2022; 82:154–160. doi: 10.1136/ard-2022-222849
